# The implication of targeting PD-1:PD-L1 pathway in treating sepsis through immunostimulatory and anti-inflammatory pathways

**DOI:** 10.3389/fimmu.2023.1323797

**Published:** 2023-12-13

**Authors:** Yu Chen, De-zhi Guo, Cheng-long Zhu, Shi-chun Ren, Chen-yan Sun, Yi Wang, Jia-feng Wang

**Affiliations:** ^1^ School of Basic Medicine, Naval Medical University, Shanghai, China; ^2^ Faculty of Anesthesiology, Changhai Hospital, Naval Medical University, Shanghai, China

**Keywords:** sepsis, PD-1/PD-L1, immune homeostasis, immunoparalysis, organ damage, treatment

## Abstract

Sepsis currently remains a major contributor to mortality in the intensive care unit (ICU), with 48.9 million cases reported globally and a mortality rate of 22.5% in 2017, accounting for almost 20% of all-cause mortality worldwide. This highlights the urgent need to improve the understanding and treatment of this condition. Sepsis is now recognized as a dysregulation of the host immune response to infection, characterized by an excessive inflammatory response and immune paralysis. This dysregulation leads to secondary infections, multiple organ dysfunction syndrome (MODS), and ultimately death. PD-L1, a co-inhibitory molecule expressed in immune cells, has emerged as a critical factor in sepsis. Numerous studies have found a significant association between the expression of PD-1/PD-L1 and sepsis, with a particular focus on PD-L1 expressed on neutrophils recently. This review explores the role of PD-1/PD-L1 in immunostimulatory and anti-inflammatory pathways, illustrates the intricate link between PD-1/PD-L1 and sepsis, and summarizes current therapeutic approaches against PD-1/PD-L1 in the treatment and prognosis of sepsis in preclinical and clinical studies.

## 1 Introduction

Sepsis is one of the leading causes of death in intensive care units (ICU) (1). In 2017, there were approximately 48.9 million globally reported cases of sepsis, resulting in a mortality rate of 22.5% and accounting for nearly 20% of all-cause mortality worldwide (2). While advancements in sepsis treatments such as antibiotics and fluid resuscitation have led to a reduction in mortality rates over the past few decades, there is still ample room for improvement (3). Furthermore, even sepsis survivors face a notable mortality risk after discharge, with rates as high as 15% within the first year (4). These statistics underscore the insufficiency of current treatments and emphasize the need to enhance our understanding of sepsis’s etiology and progression (5).

Sepsis is recognized as an immune dysregulation to various infections (6). This dysregulation manifests as a simultaneous presence of excessive inflammatory response and persistent immune paralysis (7–9). Although the patient’s immune status fluctuates, immune paralysis emerges from the onset of sepsis and serves as a critical factor contributing to multiple organ dysfunction syndrome (MODS). The programmed cell death 1 (PD-1) and its ligand PD-L1 pathway play a pivotal role in sepsis occurrence, development, and prognosis from various perspectives and levels. A considerable number of researches have demonstrated the correlation between PD-1/PD-L1 expression level and sepsis mortality, but the range of cells covered in previous articles was not comprehensive, and some were not sufficiently in-depth because the topic was not so focused (5, 10–13).

Different from those studies mentioned above, this review explores the role of PD-L1 in immunostimulatory and anti-inflammatory pathways, elucidates the intricate relationship between PD-L1 and the pathogenesis, development, and prognosis of sepsis, and summarizes current therapeutic approaches to PD-L1 in the treatment and prognosis of sepsis in preclinical and clinical studies, especially with a particular focus on PD-L1 expressed on neutrophils.

## 2 The significance of the PD-1/PD-L1 pathway in immune homeostasis

PD-1 (CD279) is a co-inhibitory receptor expressed in various locations, including the spleen, lymph nodes, bone marrow cells, and immature immune cells like CD4^+^CD8^+^T cells (11, 14–16). As a ligand for PD-1, PD-L1 (CD274) is widely expressed in hematopoietic cells and non-hematopoietic healthy tissue cells such as vascular endothelial cells and astrocytes (10). Another ligand of PD-1 is PD-L2 (CD273), which is mainly expressed in DCs and macrophages, but this review will not cover it in detail (17). Both PD-1 and PD-L1 belong to the type I transmembrane immunoglobulin (Ig) superfamily and interact via their extracellular domains, leading to a conformational change in PD-1. This prompts Src-family kinases to phosphorylate the inhibitory motif (ITIM) and switching motif (ITSM), attracting Src homology-2 containing protein tyrosine phosphatase 2 (SHP-2) and SHP-1 protein tyrosine phosphatases (9). SHP-2 dephosphorylates phosphatidylinositol 3 kinase (PI3K), thereby inhibiting Akt and ERK/MAPK signaling pathways. In the absence of SHP-2, SHP-1 acts as a compensator. Simultaneously, CD28 co-stimulatory receptors are also dephosphorylated, resulting in the inhibition of T lymphocyte activation (18–22).

The expression of PD-1 on different cells is regulated by a variety of factors. In T cells, PD-1 is increasingly expressed after antigen activation (16, 23). If the antigen is promptly eliminated, the PD-1 expression level on responding T cells decreases; otherwise, it remains elevated (24, 25). The expression of PD-1 on T cells is regulated by various factors, including activated T nuclear factor (NFAT), cytoplasm 1, Recombinant Forkhead Box Protein O1(FOXO1), T-bet, B lymphocyte-induced Maturation protein 1 (BLIMP-1), and serine-threonine kinase glycogen synthetase kinase 3 (GSK3) (26, 27). T cell receptor (TCR) activation is the primary controlling factor for PD-1 expression in T cells. Similarly, PD-L1, which is generally expressed in a variety of cells in an inflammatory environment, is regulated by multiple factors. Pro-inflammatory signals promote PD-L1 expression, in which interferon γ (IFN-γ) is considered the most effective soluble inducer (28). Also, noncoding RNAs are responsible for post-transcriptional regulation (28, 29). Additionally, protein cycling, ubiquitination, and glycosylation all influence PD-L1 expression levels (27, 30).

PD-1/PD-L1 plays a critical role in maintaining physiological health by downregulating inflammatory responses and restoring immune system balance. The interaction of PD-1 and PD-L1 facilitates autoimmune tolerance (31, 32). Reduction or deficiency of PD-L1 and PD-1 may result in species-specific autoimmunity (33, 34). Nishimura et al. found that IgG3 deposition in C57BL/6-PD-1^(-/-)^ mice caused characteristic lupus-like proliferative arthritis and glomerulonephritis, and dilated cardiomyopathy occurred in BALB/c mice with PD-1 gene destruction (35, 36). Severe impaired myocardial contraction eventually leads to congestive heart failure and even sudden death. Wang et al. also found that PD-1 deficiency significantly increased the frequency of type 1 diabetes in mice (37). Moreover, the PD-1/PD-L1 pathway also regulates atherosclerotic inflammatory responses, as demonstrated by increased atherosclerotic lesions in animals with deficiency in low-density lipoprotein receptors and PD-L1 (38).

The interaction between PD-1 and PD-L1 is closely associated with the main mechanisms of sepsis, such as inhibiting T cells function, impairing myeloid cell function, and triggering non-immune cell death (11, 39, 40). The expression of their genes significantly increases during sepsis, and PD-L1 gene deficiency improves survival in septic patients (41). Even in sepsis survivors who develop chronic critical illness (CCI), serum sPD-L1 remains elevated (42). The role of PD-L2 in sepsis is unknown, and PD-L2 gene deletion has no significant effect on the mortality of sepsis mice (41).

## 3 The role of PD-1/PD-L1 in sepsis-induced immunosuppression

**Figure 1:**
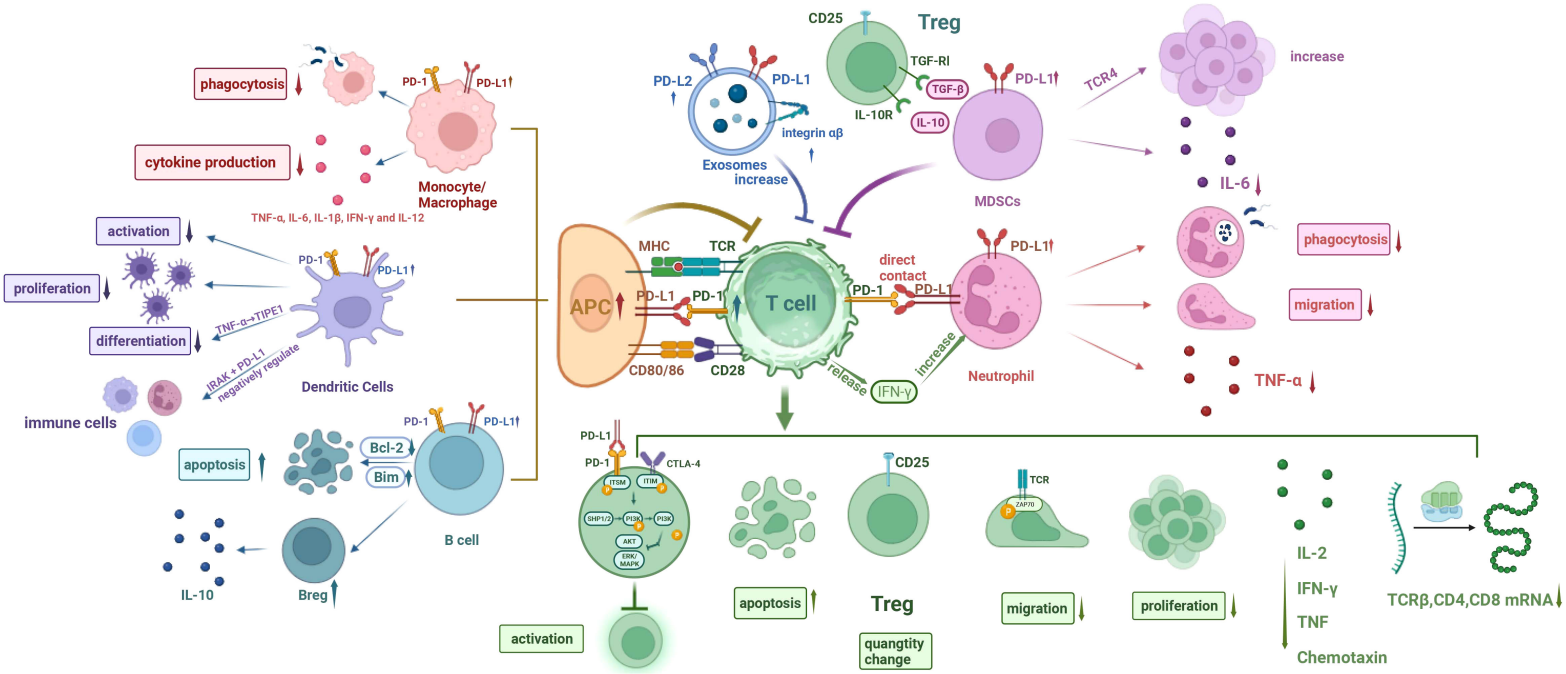
Cellular network diagram of immunoparalysis in sepsis. (I) The expression of PD-1 on T cells and PD-L1 on other immune cells are upregulated during sepsis. (II) Phagocytosis of monocytes/macrophages is compromised, and cytokine production is reduced. (III) The differentiation, proliferation, and activation of DCs are impaired, with inhibitory effect on T cell function. The endotoxin-tolerant DCs express immunosuppressive genes and have direct inhibitory effects on other immune cells. (IV) The apoptosis of B cells increases and the number of Bregs increases. (V) Increased MDSCs inhibit T cells, decrease IL-6 production, and increase the number of Tregs. (VI) Exosomal PD-L1 and PD-L1 increase and induce T cell dysfunction. (VII) The phagocytosis and migration ability of neutrophils were weakened, and TNF-α production was reduced. Neutrophils promote T cell apoptosis, inhibit T cell activation, and increase the amount of Tregs through direct contact, while IFN-γ secreted by T cells in turn increases the number of PD-L1+ neutrophils. PD-1, programmed cell death- 1; PD-L1, programmed cell death-ligand 1; MHC, major history complex; TCR, T cell receptor; CTLA-4, cytotoxic T-lymphocyte associated protein 4; TGF, transforming growth factor; IL-6, interleukin 6; TNF-α, tumor necrosis factor α; IFN-γ, interferon γ; IRAK, interleukin receptor-associated kinase; ITSM, immunoreceptor tyrosine-based switching motif; ITIM, immunoreceptor tyrosine-based inhibitory motif; SHP1/2, Src homology-2 containing protein tyrosine phosphatase 1/2; PI3K, phosphatidylinositol 3 kinase; AKT (PKB), protein kinase B; ERK, extracellular regulated protein kinases; MAPK, mitogenactivated protein kinase; APC, antigen-presenting cell; DCs, dendritic cells; Bcl-2, B lymphoblastoma-2 gene.

The definition of sepsis has been revised to refer to life-threatening organ dysfunction caused by the host’s dysfunctional immune response to various microbial infections, rather than solely excessive inflammation (43). Numerous clinical studies over the past few decades have attempted to target various mediators, such as pattern recognition receptors (PRRs), pathogen-associated molecular patterns (PAMPs), and cytokines, to suppress excessive inflammation in sepsis (44–46). However, none of these studies have yielded clinically valid results, leading to a shift in research focus. For instance, administering a single dose of tumor necrosis factor receptor-FC (TNFR-Fc) fusion protein to 141 patients with septic shock not only failed to improve patient outcomes but also increased mortality in a dose-dependent manner (47). Additionally, patients with sepsis are more prone to nosocomial infections, indicating the possible presence of persistent immunosuppression (48–52). While the clinical manifestations of immune response disorders may vary, immune paralysis occurs early in sepsis and persists due to innate and acquired immune dysfunction (**Figure 1**) (9, 53).

Studies have found that the expression of both PD-1 and PD-L1 is upregulated on T cells and monocytes respectively (54–57). During early sepsis, activation of the PD-1/PD-L1 pathway leads to innate immune cell dysfunction, and treatment with anti-PD-L1 antibodies can reverse monocyte dysfunction and inhibit T-cell apoptosis (56). Studies have demonstrated that mice deficient in PD-1 or PD-L1 exhibit improved survival during early sepsis (54). Moreover, elevated PD-L1 expression in neutrophils is associated with a higher risk of sepsis-related mortality due to increased expression of inflammatory cytokines (57). In advanced sepsis, therapy with anti-PD-1 antibodies reactivates antigen-presenting cells and T cells, thereby mitigating secondary infections (58, 59).

### 3.1 T cells

Multiple studies have shown that the PD-1/PD-L1 pathway plays a significant role in the immunosuppression seen in sepsis (60). The expressions of PD-1 on T cells and PD-L1 on macrophages and endothelial cells increase during advanced sepsis (60). Interfering with this pathway can restore T-cell activity (61, 62). In a mouse model of Candida auris infection, it was found that the positive rate of PD-1 on T cells and the frequency of PD-L1 positive macrophages were significantly higher in infected mice compared to uninfected mice (63). Additionally, the PD-L1 expression level was strongly positively correlated with the fungal tissue load. In patients with severe corona virus disease 2019 (COVID-19), progressive lymphocytopenia and depletion of lymphocyte subsets were observed, and PD-1 expression on CD4^+^ and CD8^+^ T cells was significantly increased in patients with poor prognosis (64).

In addition to the aforementioned T cell activation disorders, the depletion of T lymphocytes by the PD-1/PD-L1 pathway is also a possible internal mechanism (65–68). In sepsis, T cells are constantly exposed to antigens and inflammatory signals, leading to gradual depletion and loss of effector function, and the long non-coding RNA HOTAIRM1 may be the culprit (69). Initially, T cells experience a reduction in their proliferative capacity and production of interleukin 2 (IL-2) (70), which is followed by decreased production of IFN-γ, TNF, and chemokines, ultimately resulting in immune paralysis (70). Lymphocyte activation gene 3 (LAG3) and PD-1 have a potential synergistic effect in regulating the progressive depletion of T cells in sepsis (71). Furthermore, Zinselmeyer et al. discovered that during persistent viral infection, immune paralysis is anatomically localized in the limbic region of the spleen/red marrow and characterized by drawn-out motor paralysis of virus-specific T cells (72). Planar bilayer data indicated that localized PD-L1 within the central supramolecular activation cluster (cSMAC) can hinder the movement of CD8^+^ T cells and promote stable p-ZAP-70 immune synapse formation (73). Moreover, soluble PD-L1 was negatively correlated with TCRβ, CD4, and CD8 mRNA (74). Moreover, phosphatidylcholine (PC), 2-ethyl-2-hydroxybutyric acid, and glyceraldehyde can also be used to modulate the PD-1 expression on CD4^+^ T cells with the help of related environmental factors such as IL-2 or Lac, thus affecting the 7-day prognosis of septic patients (75).

Under physiological conditions, regulatory T cells (Tregs) maintain self-tolerance by inhibiting the activation of autoreactive T cells. And Tregs also generate pathologic immunosuppression in sepsis by suppressing the functionality of specific immune cells (76). Previous research showed that, during sepsis, there was an increase in the number of Tregs, resulting in immune paralysis by inhibiting the function of effector T cells, monocytes, and neutrophils (76).

### 3.2 B cells

Umakoshi et al. observed a significant increase in bone marrow cells of mice and a decrease in circulating B cells 6 hours after cecal ligation and puncture (CLP) (77). Additionally, CD5-expressing regulatory B cells (B regs) were found to emerge and secrete IL-10, with increased levels of IL-10 and PD-L1 mRNA detected in the spleen. In the later stages of sepsis, the expression of the B lymphoblastoma-2 gene (Bcl-2) in the spleen gradually declined, while the pro-apoptotic protein Bim showed an obvious increase. These findings suggest that B-lymphocytopenia accompanied by the increase of B regs occurs early in sepsis and may contribute to immune paralysis during septic conditions. In addition, PD-1/PD-L1 may be involved with CD72/CD100 in the formation of immune disorders during human immunodeficiency virus (HIV)-1 infection. In addition to HIV-1-specific T-cell dysfunction, it was also observed that PD-1/PD-L1 and CD72/CD100 markers on B cells were significantly enhanced during active HIV-1 infection (78).

### 3.3 Neutrophils

**Figure 2:**
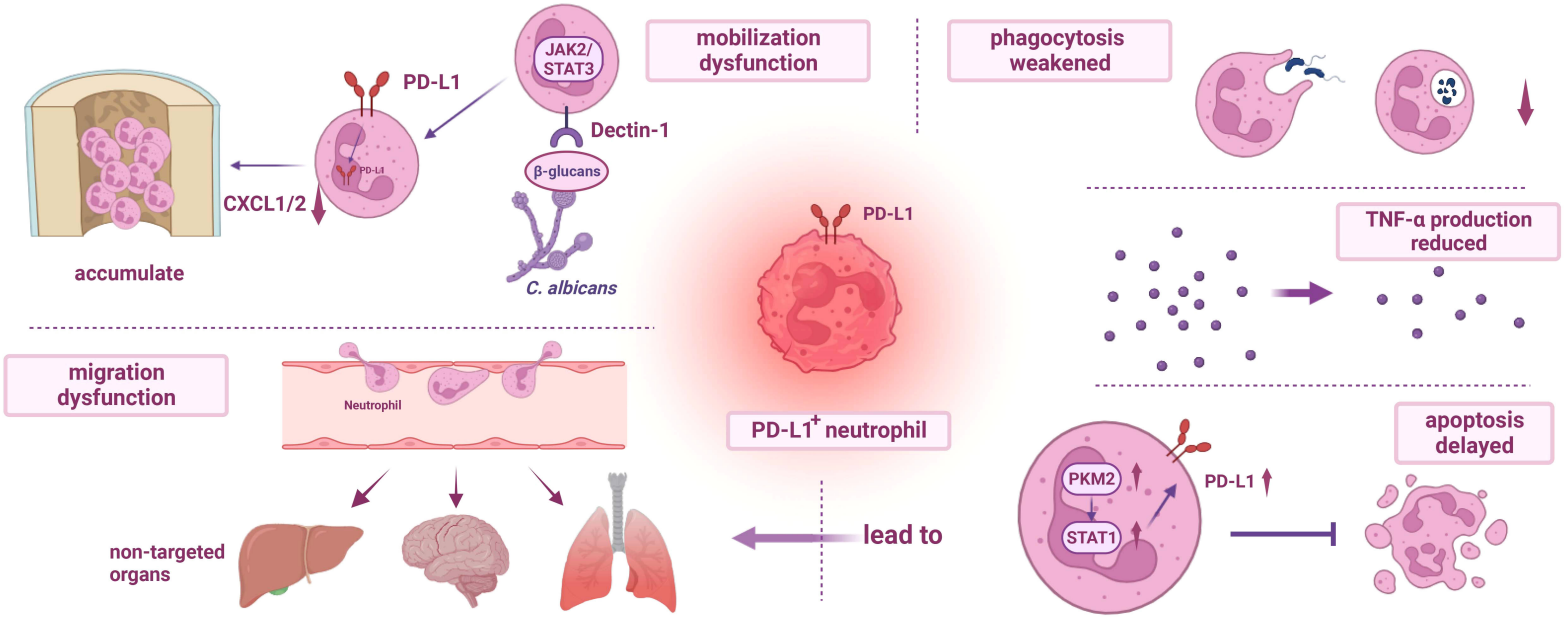
Dysfunction of PD-L1+ neutrophils. (I) The mobilization of PD-L1+ neutrophils is impaired. During fungal infection, β-glucans activate Dectin-1 and regulate the production of CXCL1/2, resulting in the accumulation of neutrophils in the bone marrow and further weakening the host immunity. (II) The migration dysfunction of PD-L1+ neutrophils and delayed apoptosis mediated by PKM2/STAT1 lead to excessive accumulation of neutrophils in non-targeted organs, resulting in organ damage. (III) Phagocytosis of PD-L1+ neutrophils is impaired, and the release of TNF-α is reduced. PD-L1, programmed cell death-ligand 1; JAK2, janus kinase 2; STAT3, recombinant signal transducer and activator of transcription 3; PKM2, recombinant pyruvate kinase M2; CXCL1/2, chemokine (C-X-C motif) ligand 1/2; TNF-α, tumor necrosis factor α.

It is well-established that neutrophils undergo phenotypic, functional, and morphological changes in the circulatory system several hours after sepsis onset, and the upregulation of PD-L1 expression may play a key role in this process (**Figure 2**) (74, 79–82). Under physiological conditions, neutrophils eliminate pathogenic microorganisms through adhesion, migration, phagocytosis, and respiratory eruption (83). However, in sepsis, neutrophils have abnormal mobilization, delayed apoptosis, migration dysfunction, phagocytosis dysfunction, and other abnormal manifestation (84–88). At the same time, neutrophils also inhibited the function of lymphocytes through contact and non-contact inhibition (89, 90).

Research has confirmed that PD-L1 expression on neutrophils increases during sepsis through the p38α−MSK1/−MK−2 pathway, and neutrophils migrate from the bone marrow to the blood and peritoneal cavity (81, 90). Patera et al. found that during sepsis, while the expression of PD-L1 was up-regulated, the ability of neutrophils to phagocytose bacteria was decreased, and the production of TNF-α was reduced, which may be related to immune paralysis (91). The impaired migration ability of PD-L1 positive neutrophils might also be associated with sepsis-induced immune paralysis, and septic neutrophils can induce lymphocyte apoptosis through direct contact, which can be reversed by anti-PD-L1 antibodies (81, 90). In addition, neutrophils can also inhibit T cell activation, promote T cell differentiation, and increase Tregs through direct contact (81). Yu et al. also found that during fungal sepsis of Candida albicans infection, PD-L1 expression is upregulated on mouse and human neutrophils. PD-L1 translocates into the nucleus under β-glucan-induction, modulates the production of chemokine (C-X-C motif) ligand 1/2 (CXCL1/2), and eventually leads to the accumulation of bone marrow neutrophils, weakening the host’s anti-infection ability (92). At the same time, IFN-γ secreted by T lymphocytes induces the production of polymorphonuclear leukocytes (PMN) with high PD-L1 expression via the JAK2/STAT1 pathway, and the Jak2 inhibitor fedratinib has been shown to significantly reduce PD-L1 expression levels in neutrophils (93). This suggests that the upregulation of PD-L1 expression on neutrophils, interacting with T cells, plays a critical role in immune paralysis. Both the p38α−MSK1/−MK−2 pathway and the JAK2/STAT1 pathway mentioned above have an impact on the expression of PD-L1 in neutrophils, but it is difficult to say which one is more important because the regulation of intracellular signal transduction often has a complex interactive network and many life activities are not controlled by a single signaling pathway. However, it has been found that the signal transduction of JAK-STAT1 may also depend on the activity of p38, and there may be a p38-JAK-STAT1 axis, so more studies need to be carried out to further explore the underlying mechanism (94). In addition, PD-L1-positive neutrophils have the potential to predict and diagnose sepsis (95).

### 3.4 Monocytes/Macrophages

Studies have shown that PD-1 and PD-L1 expression on mononuclear/macrophage increases at 24 hours of CLP treatment in mice (56). Macrophage phagocytosis and stimulus-induced cytokine production (TNF-α, IL-6, IL-1β, IFN-γ, and IL-12) were significantly decreased after CLP treatment, but random migration and cell diffusion were enhanced (54, 96). PD-1 on T cells and PD-L1 on macrophages are significantly up-regulated in mice infected with Candida auris AR-0384 (63). Shikonin can reduce PD-L1 expression on macrophages through phosphorylation of recombinant pyruvate kinase M2 (PKM2) and downregulation of nuclear input, and PKM2 can bind to the hormone receptor enzyme-1(HRE-1) and HRE-4 sites of the PD-L1 promoter (97). In addition, blood oxygen saturation at admission can be used as an effective predictor of monocyte PD-L1 expression level and immune response impairment (98). The ratio between the total level of activated CD86^+^ macrophages and the PD-1^+^ population in CLP mice was also associated with susceptibility to secondary fungal infection (99).

### 3.5 Dendritic cells

Previous studies have shown that in the early stage after CLP treatment, spleen DCs are mainly activated, and in the late stage, negative co-stimulatory molecules PD-L1 and PD-1 expression are up-regulated (100, 101). Their upregulation inhibits the activation and proliferation of DCs and affects the activation of T cells, thereby inducing immunosuppression (102). Tumor necrosis factor α-induced protein-8-like 1 (TIPE1) may play a negative regulatory role in sepsis by inhibiting DCs maturation and T cells functionality through PD-L1/PD-1 (103). In addition, endotoxin-tolerant DCs express negative regulatory genes of inflammation and have direct regulatory effects on other immune cells, which may be mediated by Interleukin-1 Receptor Associated Kinase (IRAK)-M and PD-L1 (104).

### 3.6 MDSCs

One important mechanism contributing to immune paralysis in sepsis is the expansion of myeloid-derived suppressor cells (MDSCs) (105–107). MDSCs are a heterogeneous population of immature bone marrow cells that inhibit antigen-specific activation of CD4^+^ and CD8^+^ T cells (108). Although typically undetected, elevated levels of MDSCs are observed in both cancer and sepsis, often accompanied by an increase in regulatory T cells (109–111). Hess et al. found that the increase of Tregs is mediated by transforming growth factor β (TGF-β) and IL-10 secreted by MDSCs (112). In addition, MDSCs may play a key inhibitory role on T cells through the PD-1/PD-L1 axis (53). Active Toll-like receptor 4 (TLR4) can induce monocyte MDSC expansion, thereby impacting antigen-specific T-cell initiation and IgG production (113). Clinical data analysis indicates a correlation between increased blood MDSC levels and a higher prevalence of nosocomial infections in septic patients (114). Ao et al. demonstrated that the Gr-1^hi^ cells mediated by PD-1/PD-L1 is crucial for the development of immune paralysis in later stages of sepsis (115). The Gr-1^hi^ cells were identified as MDSCs and exhibited a polymorphonuclear phenotype expressing CD11b and Ly6G markers (116). Following the injection of lipopolysaccharide (LPS) into mice treated with zymosan (ZM) on day 21, serum IL-6 production was reduced, while CD11b^+^ Gr-1^hi^ cells accumulated in the peripheral blood (115). Additionally, transferring Gr-1^hi^ cells to control mice decreased IL-6 production, but this inhibitory effect was not observed in PD-1/PD-L1-deficient d21-ZM mice. Conversely, treatment with anti-GR-1 monoclonal antibody (mAb) or anti-PD-1 and anti-PD-L1 mAb improved ZM-induced immune paralysis during sepsis induction. These findings suggest that the accumulation of Gr-1^hi^ cells mediated by PD-1/PD-L1 is another critical contributor to immune paralysis in sepsis.

### 3.7 Exosomes

Studies have revealed that exosomes (EVs) play a crucial role in tumor-associated immune paralysis by acting as carriers for PD-L1 on PD-1 and inducing potent inhibitory signals (117–119). Recently, the involvement of EVs in the pathogenesis of sepsis has also attracted attention (120). EVs are lipid bilayer nanoparticles containing RNA, DNA, and proteins (121). Studies have shown that normal cells or tumor cells release exosomes through exocytosis, which are multivesicular body (MVB) formed by inward budding of vesicles in the late endosome, and PD-L1 on the cell surface enters MVB during endocytosis (122). Huang et al. found that the expression level of circulating EVs in septic patients was higher than that in healthy controls, and it inhibited T cell function in a concentration-dependent manner, which was represented by significantly reduced expression of CD69, up-regulated expression of PD-1 and increased proportion of Treg, which may be one of the mechanisms leading to immunosuppression of sepsis (119). Kawamoto et al. reported the presence of PD-L1 and PD-L2 on circulating exosomes in the plasma of septic patients and found elevated levels of β2 integrin and PD-L2 on exosomes in these patients (123). Although there was no significant difference in PD-L1 levels on exosomes, the overall concentration of sPD-L1 increased. Furthermore, the expression levels of sPD-L1 and leukocyte β2 integrin were strongly associated with organ dysfunction. Further investigation is warranted to explore the role of exosomal PD-L1 and PD-L2 in sepsis-induced immune paralysis.

## 4 Role of PD-L1-mediated intracellular signaling in sepsis-induced inflammatory organ injury

Sepsis is a condition that can result in multiple organ failure, leading to fatal outcomes, and the interaction of PD-1/PD-L1 with CD80, PI3K/Akt pathway, or STAT1 molecules plays an important role in the mechanism of sepsis-induced organ dysfunction as described below (124, 125).

### 4.1 Lung

The lung is so vulnerable to sepsis that respiratory dysfunction has been reported to almost 70% (126). And sepsis-induced lung damage is a leading cause of mortality in patients (127, 128). In a study investigating lung injury in neonatal sepsis, Fallon et al. observed significant increases in pulmonary edema (PE), neutrophil infiltration, myeloperoxidase (MPO) levels, and cytokine expression in wild-type (WT) mice 24 hours after treatment with cecal serous fluid (CS) (129). Neonatal mice lacking PD-1 (PD-1^−/−^) showed improved survival rates compared to WT mice, particularly with noticeable differences in lung damage. However, the survival rate of neonatal mice lacking PD-L1 (PD-L1^−/−^) did not show significant improvement. Additionally, the number of PD-1^+^ cells in the lungs of human newborns with intrauterine infection was prominently higher than those who died from non-infectious causes (129). Therefore, PD-1 plays a pivotal role in the mechanism of sepsis-induced lung injury, and PD-1/PD-L1 inhibitors may be potential therapeutic targets (130). Alfred Ayala et al. constructed a mouse model of sepsis-induced lung injury using septic aggression-induced sepsis after hemorrhagic shock (Hem-CLP) and found that PD-L1 expression was significantly upregulated on vascular endothelial cells (ECs) or lung epithelial cells (EpiCs) in mice with indirect acute lung injury (iALI) 24 hours after sepsis injury (131). Moreover, inhibiting PD-L1 expression on ECs using PD-L1 siRNA encapsulated by liposomes inhibited the iALI-induced increase in cytokine/chemokine levels, as well as pulmonary myeloperoxidase and caspase 3 activities. This treatment also preserved normal tissue structure, alleviated pulmonary edema, and reduced neutrophil influx caused by iALI. However, inhibiting PD-L1 expression on EpiCs through endotracheal administration did not yield the same effects. Thus, it can be concluded that ECs, but not EpiCs, play a significant role in sepsis-induced lung injury and are closely associated with PD-L1 expression. Zona occludens-1 (ZO-1), a protein found within ECs in the lungs of WT mice, is known to shift from membranous to perinuclear position 24 hours after treatment, while PD1^−/−^ mice retain the membranous position (129). In summary, both the presence or absence of the PD-1 gene and the level of PD-L1 expression on ECs have a profound impact on lung injury induced by sepsis.

Although the precise mechanisms have not yet been fully elucidated, they generally involve the following aspects. Firstly, the upregulation of PD-1 expression on immune cells and PD-L1 expression on ECs can impair the barrier function of ECs and increase monolayer permeability (132). Moreover, under TNF-α stimulation, the expression of EC connexin on EC monolayer of PD-L1^−^ mice was increased *in vitro* and EC activation was decreased through the angiopoietin/Tie2 pathway (132). Additionally, PD-L1 plays a key role in regulating the suppression of iALI by Treg cells, which may be related to the activation of SHP-1 of lung tissue (133, 134). The activation of SHP-1 is associated with the loss of the protective effect of Tregs *in vivo* (135). Equally important, PD-L1 binds to the p85 subunit of PI3K on the endoplasmic reticulum (ER) of neutrophils, inhibiting autophagy through the PI3K/Akt/mTOR pathway and promoting the release of neutrophil extracellular traps (NETs) (136). This process leads to severe acute inflammatory lung injury, including acute respiratory distress syndrome (ARDS) (136). Li et al. found that PKM2/STAT1 mediates the up-regulation of PD-L1 expression on neutrophils and its anti-apoptotic effect, which may lead to the increase of pulmonary neutrophils accumulation and promote the occurrence of lung injury (137). Additionally, Gao et al. found through *in vitro* studies that PD-L1 regulates LPS-induced inflammation in EpiCs and vascular ECs by interacting with the hypoxia-inducible factor 1α (HIF-1α) signaling pathway. Downregulation of HIF-1α can reduce PD-L1 expression, which further inhibits HIF-1α protein expression and related pathways. The exact mechanism underlying this relationship has yet to be clarified (138). In addition, Group 2 innate lymphoid cells (ILC2s) may also mediate pulmonary immune homeostasis through PD-1 (139).

### 4.2 Liver

Previous studies have demonstrated that PD-L1 expression in the liver is significantly increased at mRNA transcription and immunohistochemical levels following CLP treatment compared to sham-operated controls (140–142). When hepatitis B virus (HBV)-associated cirrhosis is complicated with severe sepsis (SS), HBV-related acute-on-chronic liver failure (HBV-ACLF) can be caused by the superposition of monocyte PD-L1 up-regulation, and monocyte PD-L1 expression can also predict the 28-day mortality of HBV-ACLF (142). Anti-PD-L1 antibodies have shown significant improvement in liver injury morphology in CLP mice by reducing glutamic pyruvic transaminase (ALT) and glutamic oxaloacetic transaminase (AST) release, as well as decreasing TNF-α, interleukin (IL)-6, and IL-10 mRNA levels in the liver after sepsis (143).

Hutchins et al. found that intrahepatic Kupffer cells can exacerbate hepatic sinusoid endothelial cell damage during sepsis by binding to PD-L1 (140). It is PKM2/STAT1 that mediates the up-regulation of PD-L1 expression of neutrophils and promotes the intrahepatic accumulation of neutrophils (137). However, recent studies have challenged the conventional hypothesis by suggesting that high expression of PD-L1 on liver cells can ameliorate liver damage and improve survival in mice with sepsis (144). Liver damage during sepsis is associated with the activation of cytotoxic T lymphocytes (CTLs), and PD-L1 serves as a co-receptor that negatively regulates T cell function (144). Downregulation of PD-L1 in hepatocytes has been observed in mouse sepsis models, and restoration of PD-L1 expression through adenovirus- and transposon-based gene transfer significantly improved survival and reduced liver injury. Therefore, administration of recombinant PD-L1 or inhibition of NADPH oxidase type 2 (NOX2) activity may offer new treatment options for sepsis (144). In conclusion, further studies are needed to elucidate the role of PD-1/PD-L1 in the pathogenesis of sepsis-induced liver injury.

### 4.3 Brain

Regarding the brain, sepsis-associated encephalopathy (SAE) is characterized by acute and long-term cognitive impairment (145–147). While enhanced PD-L1 expression following surgical brain injury (SBI) can regulate neuroimmune and inflammatory responses through PD-L1^+^ astrocytes for self-protection and promote nerve repair, the opposite effect occurs in brain injury caused by sepsis (148). Our previous data showed that during sepsis, PD-L1 binds to P-Y705-Stat3, promoting nuclear translocation of PD-L1 and enhancing the transcription of GSDMD, resulting in increased release of neutrophil extracellular traps (NETs) (149). Neutrophils and NETs contribute to blood-brain barrier breakdown in the hippocampus, neuronal apoptosis, microglia activation, and hippocampal-dependent memory impairment (150–153). Treatment with anti-Gr-1 antibodies or DNase I has been shown to attenuate these sepsis-induced changes (149).

### 4.4 Kidney

Sepsis is one of the leading causes of acute kidney injury (AKI) in the ICU, and its occurrence correlates positively with patient mortality (154–157). The pathogenesis of septic AKI is not yet fully understood. Serum sPD-L1 levels were significantly elevated in sepsis patients with impaired renal function (158). Xu et al. established a septic AKI model induced by CLP and found increased expressions of PD-1 and PD-L1 in septic AKI mice, leading to T cell apoptosis (159). Compared to the sham group, the number of lymphocytes was reduced by 64% in sepsis mice, including a 27% decrease in CD3^+^ T cells. The results also suggest that lactate upregulates PD-L1 expression in the kidney, and blocking the lactate receptor or PD-1/PD-L1 signaling may provide a novel treatment approach for septic AKI.

### 4.5 Spleen

PD-1 upregulation in the spleen is observed early in sepsis, and spleen cell apoptosis increases over time (53, 100, 160). As evident from the aforementioned studies, the persistent expansion of myeloid-derived suppressor cells (MDSCs) during sepsis is closely associated with immune paralysis, characterized by splenocyte apoptosis, decreased T cell numbers, and upregulated PD-1 expression (114). Moreover, when human ghrelin and human growth hormone (GH) are used to correct immune paralysis, a decrease in PD-1 expression is observed in the spleens of elderly rats with sepsis (161). Additionally, the administration of anti-human PD-L1 nanobody KN035 alleviates splenocyte apoptosis, as well as lung and liver damage induced by septicemia in humanized mice, ultimately improving survival (162). These findings illustrate the close association between PD-1/PD-L1 and spleen injury caused by sepsis, although the specific mechanisms require further exploration.

### 4.6 Intestines

Increased permeability of intestinal epithelia plays a vital role in the pathophysiology of numbers of gastrointestinal diseases, and its mucosal immune system plays a key role in the development and regulation of the immune system (163). Intestinal barrier dysfunction or increased intestinal permeability is a key component in the development of MODS during sepsis (164). PD-L1 is constitutively expressed in epithelial cells of the colon and stomach, contributing to the interaction between epithelial cells and lymphocytes in specific cases, participating in intestinal mucosal inflammation, and regulating intestinal immune tolerance (19). However, during sepsis, PD-L1 expression in mouse intestinal epithelial cells (IECs) is significantly increased, ileum permeability is increased, and tight junction (TJ) proteins (claudin-1, occludin, and ZO-1 proteins) are lost, resulting in severe intestinal injury (57, 165). Moreover, PD-L1 antibodies prevented the development of colitis in mice (166).

## 5 Preclinical and clinical studies targeting PD-L1 against sepsis

PD-1/PD-L1 has emerged as a critical player in the pathogenesis of sepsis, and numerous treatment approaches targeting immune checkpoints have shown a promising role of targeting PD-L1 against sepsis in preclinical and clinical studies (167–169).

### 5.1 Effectiveness of PD-L1-targeted treatments against sepsis

#### 5.1.1 Preclinical studies

Several anti-PD-L1 antibodies have been used in tumor therapy and achieved certain efficacy, including atezolizumab, avelumab, and durvalumab (55, 91, 170–173). Meanwhile, a large number of studies have shown that specific antibodies can also reverse the negative effects of high PD-1/PD-L1 expression in sepsis, both in terms of immunoparalysis and organ damage (66, 115, 136). Zhao et al. demonstrated that the anti-human PD-L1 nanobody KN035 alleviated sepsis-induced spleen cell apoptosis, as well as lung and liver injury in humanized mouse models. This led to an improvement in the overall survival rate (162).

Additionally, regulation of the expression level of PD-L1 has shown promising therapeutic potential. miR-142 can reduce CLP-induced inflammation by targeting PD-L1 in macrophages, thereby reducing sepsis (174). Shikonin, a PKM2 inhibitor extracted from a herbal medicine, also significantly decreases the PD-L1 expression on macrophages and alleviates various immune paralyzing factors through PKM2 phosphorylation and the downregulation of nuclear input (97). Farnesyl transferase inhibitor (FTI)-277, in a dose-dependent manner, downregulated PD-L1 in spleen lymphocytes of septic mice and mitigated sepsis-induced apoptosis of spleen lymphocytes with nuclear factor-κB (NF-κB) (175). The nuclear factor erythroid 2-related factor 2 (Nrf2) can interfere with the induction of PD-L1 and inhibit the expression of PD-L1 in the later stage of sepsis, to reduce the occurrence of immunosuppression in sepsis (176). Ascorbic acid prevents sepsis-induced organ dysfunction through the p-STAT1/PD-L1 signaling pathway (177). Zusanli (ST36), Guanyuan (CV4), and Qihai (CV6) acupoint electroacupuncture modulated the immune function of sepsis patients through the PD-1/PD-L1 pathway and improved clinical symptoms (169). Furthermore, adoptive transfer of bone marrow-derived dendritic cells (BMDCs), niacinamide nucleoside supplementation, fibronectin FN C-terminal heparin-binding domain polypeptide (rhFNHC-36), glutamine (GLN), recombinant enhancer of zeste homolog 2 (EZH2) inhibitor GSK343, mitogen-activated protein kinase phosphatase 1 (Mkp-1), mycophenolate mofetil (MMF), anti-ICAM (intercellular adhesion molecule)-1 antibody and other methods have shown improved survival rates in septic mice (178–184). Notably, downregulation of PD-L1 expression has been observed in these approaches.

The involvement of neutrophils in sepsis is notable, and more and more studies showed that PD-L1 positive neutrophils were crucial for the development of inflammatory and immunological disturbance (173). The reduction in immunosuppression subsequently decreased the apoptosis rate of T lymphocytes, thereby improving the survival rate in septic mice (173). In addition, β-glucan induces an increase in the production of the chemokine CXCL1/2 by PD-L1, which leads to the accumulation of bone marrow neutrophils, weakening the host’s ability to resist fungal infection, and therefore can be used as a potential target for the treatment of fungal sepsis (92). Treatment with anti-PD-L1 antibodies or DNase I has been shown to attenuate the sepsis-induced changes in the lungs and brains caused by neutrophils (136, 149, 185).

Sepsis is more prevalent in the elderly population (186–189). The percentage of PD-1^+^ T cells and Tregs increased in elderly patients with sepsis (190). Wang et al. found that combined treatment with human growth hormone (Ghr) and human growth hormone (GH) prevented the loss of spleen T cells in elderly septic rats, thereby reducing lymphocyte apoptosis. This treatment also inhibited an increase in Treg cell number and PD-1 expression (191). They further revealed that human Ghrelin and GH can inhibit TGF-β production in a vagal-dependent manner, thus correcting immunosuppression in elderly septic rats. Treatment reduced PD-1 expression in the spleen of elderly septic rats, increased human leukocyte antigen-DR (HLA-DR) expression, alleviated lymphocyte reduction, and decreased caspase-3 levels (161, 192, 193).

#### 5.1.2 Clinical research

The area under ROC curve (AUC) of serum soluble PD-L1 (sPD-L1) combined with Sequential Organ Failure Assessment (SOFA) score is known to be of considerable value in the diagnosis of sepsis, and during the first week of ICU treatment, sPD-L1 was a valuable predictor of severe sepsis and septic shock severity and 28-day mortality (194–196). sPD-1 levels and CRP and PCT levels were positively correlated, so the correlation between sPD-1 and inflammatory markers may also serve as a potential biomarker for the diagnosis of sepsis (197).

In the therapy aspect, A Phase 1b randomized study evaluated the relevant aspects of Nivolumab (198). Nivolumab is a monoclonal antibody targeting PD-1 and it is approved for the treatment of various cancers (199–202). The study found that the pharmacokinetic profile of Nivolumab (480 mg or 960 mg) resulted in a receptor occupancy greater than >90% for at least 28 days, with no evidence of worsening symptoms such as fever, shock, or cytokine storms. Watanabe et al. found that when treated with Nivolumab, there was an observed increase in absolute lymphocyte counts and monocyte HLA-DR subtype expression levels over time (203). The incidence of adverse events in the 480 mg and 960 mg groups was reported as 80% and 50%, respectively. Notably, only one drug-related adverse event was observed in the 480 mg group, and no Nivolumab-related deaths occurred. In conclusion, a single dose of 960 mg of Nivolumab demonstrated good tolerability and maintained adequate blood concentration of the drug. Furthermore, both the 480 mg and 960 mg doses of Nivolumab appeared to improve immune system markers for the study (203). Moreover, Zusanli (ST36), Guanyuan (CV4) and Qihai (CV6) acupoint electroacupuncture can also regulate the immune function of septic patients through PD-1 pathway and improve clinical symptoms (169).

However, van den Haak et al. found that PD-1 suppression at a single high dose of 480mg or 960mg of Nivolumab lasted for more than 90 days in most cases, while the duration of sepsis was 7-10 days, so it may induce long-term immune-related side effects (204). In contrast, a single dose of 20mg of Nivolumab (median 23 days and effective) may be more appropriate as a therapeutic dose for sepsis. In addition, Hong et al. found that using selective beta-blockers (especially atenolol) improved sepsis incidence and course, significantly reduced serum sPD-L1 levels, and facilitated ROS-induced NF-κB and STAT3 activation, thus down-regulating PD-L1 expression on monocytes/macrophages (205). In addition, since PD-1 inhibitors often require modification to improve stability, poor modification may result in a higher incidence of irAEs than PD-L1 inhibitors (206). And PD-1 inhibitors block both PD-1/PD-L1 and PD-1/PD-L2 pathway simultaneously, thus reducing local homeostasis of macrophages, which may also increase the incidence of irAEs (207). But other studies have shown no significant difference, and there is currently no comparison between the two in the field of sepsis treatment (208, 209).

#### 5.1.3 COVID-19

Coronavirus disease 19 (COVID-19) is a viral sepsis characterized by lymphocytopenia, which is particularly prominent in severe cases of COVID-19. One of the main changes in these patients was the increased counts of neutrophils and decreased counts of lymphocyte, thus the neutrophil-lymphocyte-ratio is a potential marker of severity of COVID-19 (210–212). Additionally, co-inhibitory molecules such as PD-1, PD-L1, cytotoxic T-lymphocyte-associated protein 4 (CTLA-4), and T cell immunoglobulin domain and mucin domain-3 (TIM-3) are found to be overexpressed in CD4^+^ and CD8^+^ T cells, and high levels of PD-L1 expression are associated with lymphocytopenia and increased mortality in COVID-19 patients (211, 213).

Vitamin D is a potentially effective treatment for COVID-19 (214–217). It was reported that vitamin D reduced PD-L1 levels when serum PD-L1 was very high, and vice versa (218). At the same time, vitamin D can also reduce PD-L1 expression by reducing pro-inflammatory cytokines such as IL-6, TNF-γ, etc (219, 220). But vitamin D supplementation also increased the expression of PD-L1 on Tregs, increased the depletion of T cells, and worsen the immunoparalysis (221). Therefore, the use of vitamin D might be a choice in treating COVID-19.

These findings suggested the potential use of anti-PD-1/PD-L1 antibodies in COVID-19 patients with or without cancer. Currently, there are five clinical trials registered on clinicaltrials.gov aiming to investigate the efficacy of anti-PD-1 antibodies in treating COVID-19 (NCT04333914, NCT04268537, NCT04356508, NCT04343144, NCT04413838). These studies include patients with metastatic and advanced cancer who have also been affected by COVID-19, as well as obese patients with COVID-19 infection. The trials aim to assess the effectiveness of various anti-PD-1 antibodies, such as nivolumab, either in combination with standard treatment regimens (NCT04333914) or as standalone treatments (222).

### 5.2 Emerging approaches to address current treatment challenges

Despite the accumulation of preclinical evidence demonstrating the efficacy of various treatments, there is still a lack of robust clinical studies supporting their use. Immune checkpoint inhibitors (ICIs) used in cancer treatment research have been associated with immune-related adverse events (irAEs), such as rashes, colitis, thyroiditis, and pneumonia (223–225). Additionally, ICIs can also lead to systemic hyperinflammatory syndrome, although it is less common (226). Therefore, it is evident that PD-1/PD-L1-related treatments in sepsis may have similar side effects (227). The therapeutic effect was related to the immune status and initial pathogen load before treatment (228, 229). Studies have shown that anti-PD-L1 treatment failed to improve survival rates in a fatal Staphylococcus aureus pneumonia mouse model, but the exact reason is unclear (230).

To improve the positive rate of clinical trials and reduce side effects, many new ideas and attempts have been produced. For example, the above-mentioned KN035 is derived from a single-domain antibody with a lower molecular weight than normal monoclonal antibodies, which may give it more favorable physical and chemical properties, but the exact mechanism and effects are still unknown (162).

Peptide immune checkpoint antagonists present a potential alternative drug model. Unlike sustained blocking of the PD-1 pathway by antibodies, peptide-based therapies offer a rapid pharmacokinetic profile that reduces the likelihood of irAEs. In a mouse model of Candida albicans sepsis, Hotchkiss et al. evaluated the efficacy of a novel short-acting anti-PD-L1 peptide called compound 8, which demonstrated a twofold increase in survival compared to the control group (231). Gutierrez et al. also reported an effective peptide-based PD-1 checkpoint antagonist (LD01) that significantly improved survival by enhancing macrophage phagocytosis activity and T-cell production of IFN-γ (232).

The lack of success in clinical trials may be attributed to the use of animal models established using relatively uniform “inducers” in genetically homogenous strains of laboratory animals, which do not fully capture the pathophysiology of human sepsis and the heterogeneity of patient populations. Heterogeneity in patient factors such as genetic and social backgrounds, cause of sepsis, personal medical history, and disease course (233). Accurate sepsis diagnosis and clinical classification can help improve treatment efficacy to some extent. For example, early administration of Nivolumab 6mg/kg in combination with the antibiotic meropenem fully alleviated bacterial sepsis when the initial pathogen load was below 3,000 CFU/μL, but not when the initial load was above 5,000 CFU/μL (229). Artificial intelligence (AI) may also be valuable in this area. AI has shown a promising ability to predict early-stage organ dysfunction, such as acute kidney injury and ARDS, leading to improved outcomes (234). Furthermore, AI has facilitated the unprecedented classification of four sepsis subgroups based on big data analysis, which might guide a more precision clinical treatment (12).

### 5.3 Role of PD-1/PD-L1 in prognostic prediction

Besides its therapeutic effects, PD-1/PD-L1 pathway is also promising in prognostic prediction both in terms of acquired immune cells and innate immune cells (235, 236). Li et al. conducted a two-phase cohort study to assess the predictive effect of PD-1 on 28-day mortality in sepsis patients (237). The analysis included a total of 120 patients, with 58 patients in phase I (test set) and 62 patients in phase II (validation set). The findings revealed that the expression of PD-1 in Tregs and the Sequential Organ Failure Assessment (SOFA) score were independent risk factors for 28-day mortality. Moreover, the expression of PD-1 on CD4^+^ and memory CD8^+^ T cells and the PD-1/CD28 ratio in CD8^+^ T cells are also significantly correlated with the severity and prognosis of sepsis patients (238–241). Furthermore, Zeng et al. conducted a cohort study involving 114 patients, demonstrating that the percentage of PD-L1^+^ NK cell and the SOFA score were independent risk factors for 28-day mortality (242). PD-L1 expression levels on monocytes, DCs, and neutrophils combined with SOFA or APACHE II scores have also been used to predict sepsis mortality (90, 142, 243–245). Overall, the evaluation of PD-1 and PD-L1 expression in immune cells is promising in prognostic prediction for sepsis patients. These markers, along with traditional factors such as SOFA score, provide valuable insights into patient outcomes, allowing for tailored treatment strategies and improved clinical decision-making.

## 6 Conclusions

PD-1/PD-L1 is known to play a crucial role in the occurrence and development of sepsis, affecting the functionality of various immune cells and the release of immune factors, which leads to dual functional abnormalities in innate and acquired immunity (241, 246, 247). During sepsis, the expression levels of PD-1/PD-L1 on T lymphocytes, B lymphocytes, neutrophils, macrophages, myeloid suppressor cells, and exosomes show significant differences compared to the control group, resulting in immunoparalysis (248–250). PD-1/PD-L1 may mediate the damage and dysfunction of organs such as the lung, liver, brain, kidney, spleen, and intestines in sepsis, although the specific mechanism requires further investigation (141, 251). While many preclinical and preliminary studies (**Table 1**) on PD-1/PD-L1 have shown promising results, large-scale clinical studies are warranted to confirm its therapeutic effect against sepsis (257).

**Table 1:** Existing sepsis clinical studies (PD-1/PD-L1 related).

NO.	Study type	Research objective	Intervention mode	Registration number	Phase	Sample size*	Reference
1	Intervention study	treatment	Nivolumab	JapicCTI-173600	1/2	15	(203)
2	Intervention study	treatment	Nivolumab	NCT02960854	1b	31	(198)
3	Intervention study	treatment	GNS561, monalizumab, avdoralimab	NCT04333914	II	19	/
4	Intervention study	treatment	PD-1 blocking antibody+ standard treatment, Thymosin+ standard treatment	NCT04268537	II	120	/
5	Intervention study	treatment	Nivolumab	NCT04356508	II	15	/
6	Intervention study	treatment	Nivolumab	NCT04343144	II	92	/
7	Intervention study	treatment	Nivolumab	NCT04413838	II	120	/
8	Observational study	Prediction	/	/	/	91 + 29	(236)
9	Observational study	Prediction	/	/	/	120	(237)
10	Observational study	Prediction	/	2020ZDSYLL041-Y01	/	30	(240)
11	Observational study	Prediction	/	2021583	/	48 + 20	(241)
12	Observational study	Prediction	/	NCT02188992	/	406	(252)
13	Observational study	Prediction	/	/	/	210	(253)
14	Observational study	Prediction	/	/	/	70 + 17	(142)
15	Observational study	Prediction	/	/	/	177	(244)
16	Observational study	Prediction	/	/	/	114	(242)
17	Observational study	Prediction	/	/	/	28 + 10	(254)
18	Observational study	Prediction	/	/	/	62	(255)
19	Observational study	Prediction	/	/	/	118 + 21	(256)
20	Observational study	Prediction	/	/	/	135 + 29	(243)
21	Observational study	Precaution	/	EC1011008-E	/	64,070 + 64,070	(205)
22	Observational study	Diagnosis	/	201349	/	64 + 30	(195)

This table lists the current clinical research in sepsis that focuses on PD-1/PD-L1. Of them, 7 were intervention studies to investigate PD-1/PD-L1-focused sepsis treatment, 13 were for the clinical predictive effect of PD-1/PD-L1 on sepsis prognosis, and there were also preventive and diagnostic studies.

*The sample size is expressed in the form of A+B, where A represents the number of sepsis patients involved in the experiment and B represents the number of the healthy control group. If there is only one number, it means that the study does not mention the healthy control group.

## Author contributions

YC: Writing – original draft, Writing – review & editing. DG: Writing – original draft, Writing – review & editing. CZ: Conceptualization, Writing – original draft. SR: Writing – review & editing. CS: Writing – original draft. YW: Conceptualization, Supervision, Writing – review & editing. JW: Conceptualization, Funding acquisition, Supervision, Writing – review & editing.
